# Altered Lipidome Composition Is Related to Markers of Monocyte and Immune Activation in Antiretroviral Therapy Treated Human Immunodeficiency Virus (HIV) Infection and in Uninfected Persons

**DOI:** 10.3389/fimmu.2019.00785

**Published:** 2019-04-16

**Authors:** Emily R. Bowman, Manjusha Kulkarni, Janelle Gabriel, Morgan J. Cichon, Kenneth Riedl, Martha A. Belury, Jordan E. Lake, Brian Richardson, Cheryl Cameron, Mark Cameron, Susan L. Koletar, Michael M. Lederman, Scott F. Sieg, Nicholas T. Funderburg

**Affiliations:** ^1^Division of Medical Laboratory Science, School of Health and Rehabilitation Sciences, Ohio State University, Columbus, OH, United States; ^2^Personalized Food and Nutritional Metabolomics for Health Discovery Theme, The Ohio State University, Columbus, OH, United States; ^3^Department of Human Sciences, Ohio State University, Columbus, OH, United States; ^4^Division of Infectious Diseases, Department of Medicine, The University of Texas Health Science Center, Houston, TX, United States; ^5^Department of Population and Quantitative Health Sciences, Case Western Reserve University, Cleveland, OH, United States; ^6^Department of Nutrition, Case Western Reserve University, Cleveland, OH, United States; ^7^Division of Infectious Diseases, Department of Medicine, Ohio State University, Columbus, OH, United States; ^8^Division of Infectious Diseases, Department of Internal Medicine, Case Western Reserve University/University Hospitals of Cleveland, Cleveland, OH, United States

**Keywords:** lipidome, free fatty acids, HIV, monocytes, inflammation, cardiovascular disease

## Abstract

**Background:** HIV infection and antiretroviral therapy (ART) have both been linked to dyslipidemia and increased cardiovascular disease (CVD) risk. Alterations in the composition of saturated (SaFA), monounsaturated (MUFA), and polyunsaturated (PUFA) fatty acids are related to inflammation and CVD progression in HIV-uninfected (HIV–) populations. The relationships among the lipidome and markers of monocyte and immune activation in HIV-infected (HIV+) individuals are not well understood.

**Methods:** Concentrations of serum lipids and their fatty acid composition were measured by direct infusion-tandem mass spectrometry in samples from 20 ART-treated HIV+ individuals and 20 HIV– individuals.

**Results:** HIV+ individuals had increased levels of free fatty acids (FFAs) with enrichment of SaFAs, including palmitic acid (16:0) and stearic acid (18:0), and these levels were directly associated with markers of monocyte (CD40, HLA-DR, TLR4, CD36) and serum inflammation (LBP, CRP). PUFA levels were reduced significantly in HIV+ individuals, and many individual PUFA species levels were inversely related to markers of monocyte activation, such as tissue factor, TLR4, CD69, and SR-A. Also in HIV+ individuals, the composition of lysophosphatidylcholine (LPC) was enriched for SaFAs; LPC species containing SaFAs were directly associated with IL-6 levels and monocyte activation. We similarly observed direct relationships between levels of SaFAs and inflammation in HIV uninfected individuals. Further, SaFA exposure altered monocyte subset phenotypes and inflammatory cytokine production *in vitro*.

**Conclusions:** The lipidome is altered in ART-treated HIV infection, and may contribute to inflammation and CVD progression. Detailed lipidomic analyses may better assess CVD risk in both HIV+ and HIV– individuals than does traditional lipid profiling.

## Introduction

Both HIV infection and the use of antiretroviral therapy (ART) contribute to an increased risk for cardiovascular disease (CVD) (1, 2). Dyslipidemia is observed in HIV-infected (HIV+) individuals, and is associated with reduced levels of high-density lipoprotein (HDL) cholesterol, and elevated total (TC) cholesterol, low-density lipoprotein (LDL) cholesterol, and triglycerides (TG). Similar lipid abnormalities have been linked to the development of atherosclerosis in the general population (3), however, these basic lipid assessments may not sufficiently evaluate CVD risk in HIV+ individuals (4). Advances in quantitative mass spectrometry-based lipid analyses allow for more sensitive and extensive assessments of the lipidome (5).

Lipids have diverse biological roles, including signal transduction, protein trafficking, and regulation of membrane permeability (6). The physiological importance of lipids is underscored by the various diseases associated with lipid abnormalities, including CVD, obesity, diabetes, non-alcoholic fatty liver disease (NAFLD), non-alcoholic steatohepatitis (NASH), and Alzheimer's disease, many of which are increased in HIV infection (7–12). Moreover, the fatty acid composition of circulating lipids is important; increased levels of saturated fatty acids (SaFA) are associated with greater risk of diabetes, whereas increased polyunsaturated fatty acid (PUFA) levels are associated with reduced risk of CVD and mortality (13). *In vitro* exposure of myeloid cells to SaFAs activates inflammatory pathways (14); conversely, PUFAs inhibit inflammasome signaling and lipopolysaccharide (LPS)-induced endothelial cell activation (15–17).

Immune activation is characteristic of HIV infection, and is likely driven by multiple factors (18). Chronic inflammation underlies the development of many comorbidities, including atherosclerosis (19), and the inflammatory environment in HIV infection may accelerate progression of CVD. The relationship between the lipidome and inflammation is complex; lipid processing and transport is affected by inflammatory processes and many lipid species contribute to inflammation and immune activation (20). Characterizing specific alterations in the lipidome of HIV+ individuals, and their association with immune activation, may help to elucidate potential mechanisms of enhanced CVD risk and metabolic abnormalities.

We recently profiled the concentration and fatty acid composition of over 1,200 different lipid species across 13 lipid classes in HIV+ individuals pre- and post-ART exposure, and in a demographically similar HIV– population. We observed significant differences in the fatty acid composition of free (non-esterified) and lysophosphatidylcholine (LPC) fatty acids among HIV- and HIV+ individuals, and these lipidome alterations were associated with markers of immune activation in the HIV+ population. Here, we confirm broad alterations in the lipidome of ART-treated HIV+ individuals in a cross-sectional study, and further examine novel relationships among lipids and markers of inflammation and monocyte activation.

## Materials and Methods

### Study Participants

All study participants provided written informed consent in compliance with the Ohio State University (OSU) Institutional Review Board. HIV+ men currently taking suppressive ART were enrolled at the infectious disease clinic at OSU. HIV- male donors were recruited from the general population at OSU. Age, HIV-1 RNA, CD4^+^ T cell counts, duration of ART usage, TC, HDL, LDL, and TG levels, smoking status, diabetes, co-infection status, and use of statins, aspirin, and/or anti-hypertensive medications were extracted for each HIV+ participant from medical charts from the most recent time point available (date of study enrollment). Framingham risk score, developed using Framingham Heart Study data, estimates an asymptomatic patient's 10-year risk of developing CVD, and was calculated using an algorithm based on gender, age, TC, HDL, systolic blood pressure, and smoking status (21). Generally, 0–10% risk score indicates low CVD risk, 10–20% risk score indicates intermediate CVD risk, and a risk score of >20% indicates high CVD risk.

### Sample Collection

Blood samples were collected in EDTA-containing vacutainer tubes (BD Biosciences) for peripheral blood mononuclear cell (PBMC) preparation and whole blood stimulations. For serum collection, blood was drawn into 10 mL serum separating tubes (SST, BD Biosciences). SST were centrifuged for 15 min at 800 × g, and serum was collected and frozen at −80°C until thawed once and analyzed in batch.

### Cell Preparation and Culture

PBMCs were isolated by centrifugation over Ficoll-Hypaque, and cultured in RPMI 1640 supplemented with 10% autologous serum. Palmitic acid (16:0), stearic acid (18:0), and soy-derived lysophosphatidylcholine (LPCsoy) (Avanti Polar Lipids) were dissolved in 100% ethanol and filter-sterilized (10 mM stock solutions). The fatty acids, or ethanol (EtOH) vehicle control, were added (10 μM final concentration) to fresh RPMI 1640 supplemented with 5% autologous serum, and incubated for 30 min prior to stimulation of PBMCs. Cells were stimulated for 24 h with prepared fatty acid containing media, with or without LPS (100 ng/mL) (Invivogen).

For the whole blood stimulation experiments, blood (1 mL) was incubated in 15-mL conical tubes (BD Biosciences) for 3 h with individual fatty acids (10 μM final concentration for all) or ethanol vehicle control. Blood samples were then incubated for 15 min on ice with FACS Lysis buffer (BD Biosciences), and washed with flow wash buffer in preparation for analysis by flow cytometry.

### Lipid Measurement

Serum lipids were analyzed using the direct infusion-tandem mass spectrometry (DI-MS/MS) Lipidyzer platform (Sciex, MA, USA). The Lipidyzer is a validated platform for high-throughput identification and quantitation of approximately 1,100 biological lipids covering 13 lipid classes (i.e., free fatty acids, phospholipids, cholesterol esters, ceramides, sphingomyelins, diacylglycerols, triacylglycerols). An optimized sample preparation procedure and differential mobility spectrometry (DMS) are utilized for lipid separation. The Lipidyzer platform methodology has been described in detail elsewhere (22), but briefly, lipids were extracted from 100 *u*L of serum using a modified Bligh-Dyer method. Over 50 stable isotope labeled internal standards spanning all 13 lipid classes were added to each sample prior to extraction for accurate quantitation. Extracts were reconstituted in dichloromethane/methanol (1:1) and analyzed using DI-MS/MS with DMS separation. A Shimadzu LC system was used for automated infusion of each serum extract and for pumping running and rinse solutions through the lines. Serum extracts were infused into a 5500 QTRAP MS/MS with SelexION DMS technology (Sciex) and lipid species were targeted and quantitated using optimized MS/MS transitions. Data were generated using the Lipidomics Workflow Manager software (Sciex). Results provided the concentration (μM) and fatty acid composition (mol%) of total lipid classes as well as individual lipid species.

### Soluble Markers

Supernatant levels of IL-6, and serum levels of the immune activation markers soluble CD163 (sCD163), soluble CD14 (sCD14), Intercellular adhesion molecule-1 (ICAM1), and tumor necrosis factor receptor-II (TNFR-II) were measured using Quantikine ELISA kit (R&D Systems). Levels of oxidized LDL (Mercodia) and LPS-binding protein (LBP) (Hycult Biotech) were also measured by ELISA.

### Flow Cytometry

Monocytes were identified by size, granularity, and surface expression of CD14 and CD16 (Supplemental Figure 1). The following antibody-fluorochrome conjugates (and appropriate isotype controls) were used: anti-CD14 (Pacific blue), anti-CD16 (PE), anti-HLA-DR (APC-Cy7), anti-CD40 (Pe-Cy7), anti-CD86 (FITC), anti-TLR4 (Pe-Cy7), anti-CD36 (APC), anti-CD11a (Pe-Cy7), anti-CD11b (PerCP), anti-CD18 (FITC), anti-CD69 (Pe-Cy7) (BD Biosciences for all); anti-SR-A (FITC) (Miltenyi Biotec); anti-TF (FITC) (Biomedica Diagnostics). Cells were stained and then fixed with 1% paraformaldehyde, and analyzed using a Miltenyi MACSQuant flow cytometer.

### Statistical Analysis

Differences in the concentration and composition of LPC and free fatty acid levels between HIV+ and HIV– donors were assessed using unpaired Mann-Whitney tests. Associations among lipid levels and immune activation marker expression were analyzed using Spearman correlations. The statistical significance of differences in surface marker expression and IL-6 production in cells stimulated *in vitro* with various fatty acids was assessed using paired Wilcoxon tests. Data analysis was performed in GraphPad Prism 6. Due to sample size limitations, these exploratory analyses were not corrected for multiple comparisons in order to assess overall trends.

## Results

Participant demographic information (20 HIV+, 20 HIV–) is provided in Table 1. All HIV+ participants were on suppressive ART (HIV-1 RNA <40 copies/mL) for a mean of 15 years, and had a mean CD4^+^ T cell count of 603 cells/μL. Thirty percent of the HIV+ group were current smokers, 60% reported statin usage, 40% were taking anti-hypertensive medication, and 20% were taking daily aspirin. The levels of TC, HDL, LDL, and TG were not statistically different between these HIV+ and HIV– populations. Framingham risk scoring was based on a predictive algorithm to estimate an asymptomatic patient's likelihood of developing CVD within 10 years (21). The HIV+ study population had an average Framingham score of 8%, which indicates low CVD risk.

**Table 1:** Demographics and clinical characteristics of HIV– and HIV+ study participants.

	**HIV-**	**HIV+**
Age	Mean = 32 Range = 21–65	Mean = 49 Range = 18–62
HIV viral load (date of enrollment)	NR	<40
CD4+ T cell count (date of enrollment)	NR	Mean = 603 Range = 296–1010
Ethnicity (%)		
White	70	60
Non-white	30	40
Total cholesterol (mg/dL)	Mean = 176 Range = 128–254	Mean = 170 Range = 100–256
HDL (mg/dL)	Mean = 55 Range = 32–94	Mean = 50 Range = 30–101
LDL (mg/dL)	Mean = 94 Range = 25–162	Mean = 91 Range = 34–170
Triglycerides (mg/dL)	Mean = 133 Range = 45–312	Mean = 145 Range = 36–347
On ART (%)	NR	100
Duration of ART (years)	NR	Mean = 15 Range = 2–30
Current smoker (%)	NR	30
Statin use (%)	NR	60
Framingham 10-year CVD risk score (%)	NR	Mean = 8 Range = 2–28

*NR, not recorded*.

### Concentrations and Composition of Free Saturated and Unsaturated Fatty Acids Are Altered in HIV Infection

**Figure 1:**
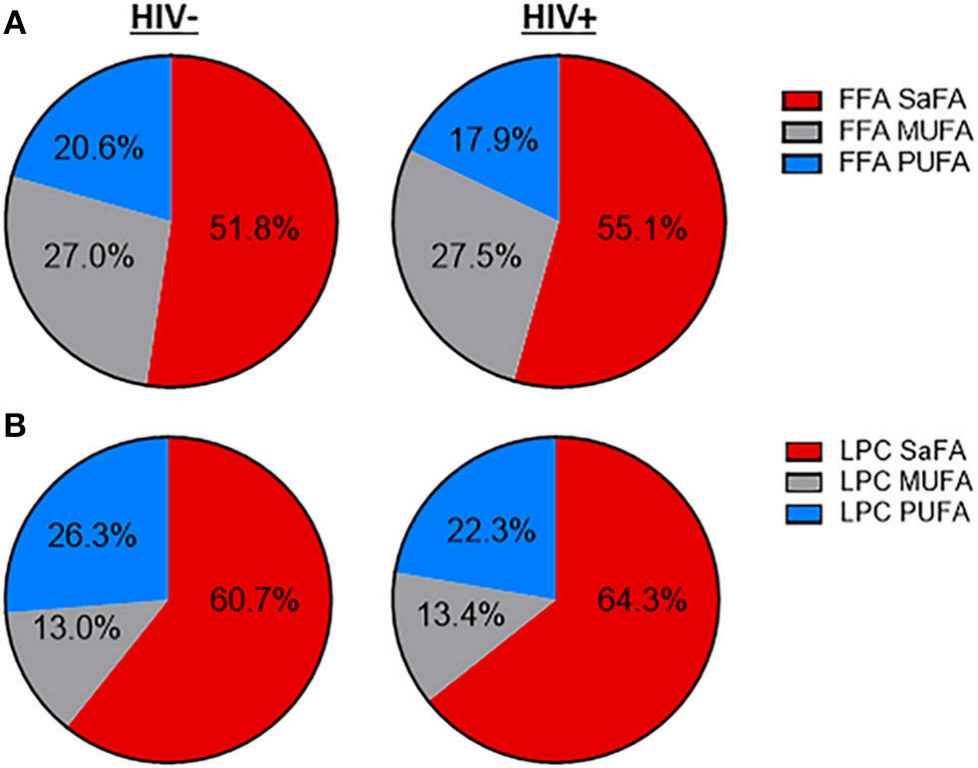
The fatty acid composition of LPC and free fatty acids is altered in treated HIV-infected individuals compared to HIV-uninfected participants. Serum lipids were analyzed using the direct infusion-tandem mass spectrometry (DI-MS/MS) Lipidyzer platform. The fatty acid composition of **(A)** free fatty acids and **(B)** lysophosphatidylcholine (LPC) are displayed.

The concentration of total free (non-esterified) fatty acids (FFAs) was greater among HIV+ participants (469.2 μM) than among HIV– participants (352.7 μM, *p* = 0.03). Concentrations of several individual free SaFAs (palmitic acid 16:0, margaric acid 17:0, stearic acid 18:0, behenic acid 22:0) were also increased significantly in the HIV+ group (Table 2). Similarly, the composition of free fatty acids differed between HIV+ and HIV– participants; the proportion of free SaFAs tended to be increased (55.1 vs. 51.8%, *p* = 0.06) and the proportion of PUFAs (17.9 vs. 20.6%, *p* = 0.03) were significantly reduced in the HIV+ participants when compared to the composition of free fatty acids (FFA) among HIV– participants (Figure 1A). HIV+ participants had significantly reduced proportions of the PUFA α-linolenic acid (18:3); proportions of dihomo-γ-linolenic acid (20:3) and arachidonic acid (20:4) also tended to be decreased in HIV+ individuals (Table 2). ART use has been linked to lipid abnormalities in HIV infection (2), and the duration of ART usage in this HIV+ study population was negatively associated with levels of free PUFAs, eicosapentanoic acid (EPA) (20:5) (*r* = −0.51, *p* = 0.03) and dihomo-γ-linolenic acid (20:3) (*r* = −0.50, *p* = 0.03) (Table 1, data not shown).

**Table 2:** Composition and concentration of free fatty acid species measured in HIV– and HIV+ individuals.

	**Composition (mol %)**			**Concentration (μm)**		
**Lipid species**	**HIV–**	**HIV+**	***p*-value**	**HIV–**	**HIV+**	***p*-value**
FFA(12:0)	**2.59**	**2.00**	**0.04**	8.83	8.06	0.33
FFA(14:0)	**2.29**	**2.76**	**0.02**	9.83	9.56	0.77
FFA(15:0)	0.88	0.79	0.26	3.04	3.21	0.45
FFA(16:0)	26.23	26.70	0.60	**92.68**	**126.68**	**0.03**
FFA(17:0)	1.20	1.18	0.87	**4.11**	**5.07**	**0.02**
FFA(18:0)	12.50	13.63	0.16	**43.49**	**65.13**	**0.03**
FFA(20:0)	0.33	0.34	0.79	1.14	1.52	0.10
FFA(22:0)	5.72	7.31	0.25	**19.83**	**26.81**	**0.04**
FFA(24:0)	**0.21**	**0.17**	**0.03**	0.73	0.69	0.64
FFA(14:1)	0.39	0.38	0.71	**1.31**	**1.58**	**0.04**
FFA(16:1)	1.84	2.01	0.52	6.56	9.86	0.09
FFA(18:1)	23.78	24.30	0.73	84.21	122.61	0.06
FFA(20:1)	0.46	0.44	0.37	1.59	2.04	0.13
FFA(22:1)	0.33	0.30	0.31	1.14	1.40	0.15
FFA(24:1)	0.24	0.25	0.78	0.85	1.16	0.12
FFA(18:2)	15.06	13.31	0.13	54.69	63.06	0.40
FFA(18:3)	**1.55**	**1.24**	**0.05**	5.57	5.68	0.92
FFA(18:4)	0.07	0.06	0.12	0.25	0.25	0.90
FFA(20:2)	0.26	0.25	0.55	0.89	1.18	0.07
FFA(20:3)	0.52	0.44	0.06	1.75	1.91	0.33
FFA(20:4)	1.94	1.57	0.07	6.64	6.77	0.87
FFA(20:5)	0.33	0.28	0.29	1.11	1.11	0.97
FFA(22:2)	0.08	0.07	0.59	0.27	0.34	0.09
FFA(22:4)	0.17	0.18	0.73	0.58	0.80	0.09
FFA(22:5)	0.25	0.22	0.15	0.88	0.99	0.43
FFA(22:6)	0.40	0.33	0.21	1.41	1.50	0.75

*The bold values indicate p-values < 0.05*.

### Free Fatty Acid Composition Is Associated With Markers of Inflammation and Monocyte Activation in HIV Infection

**Figure 2:**
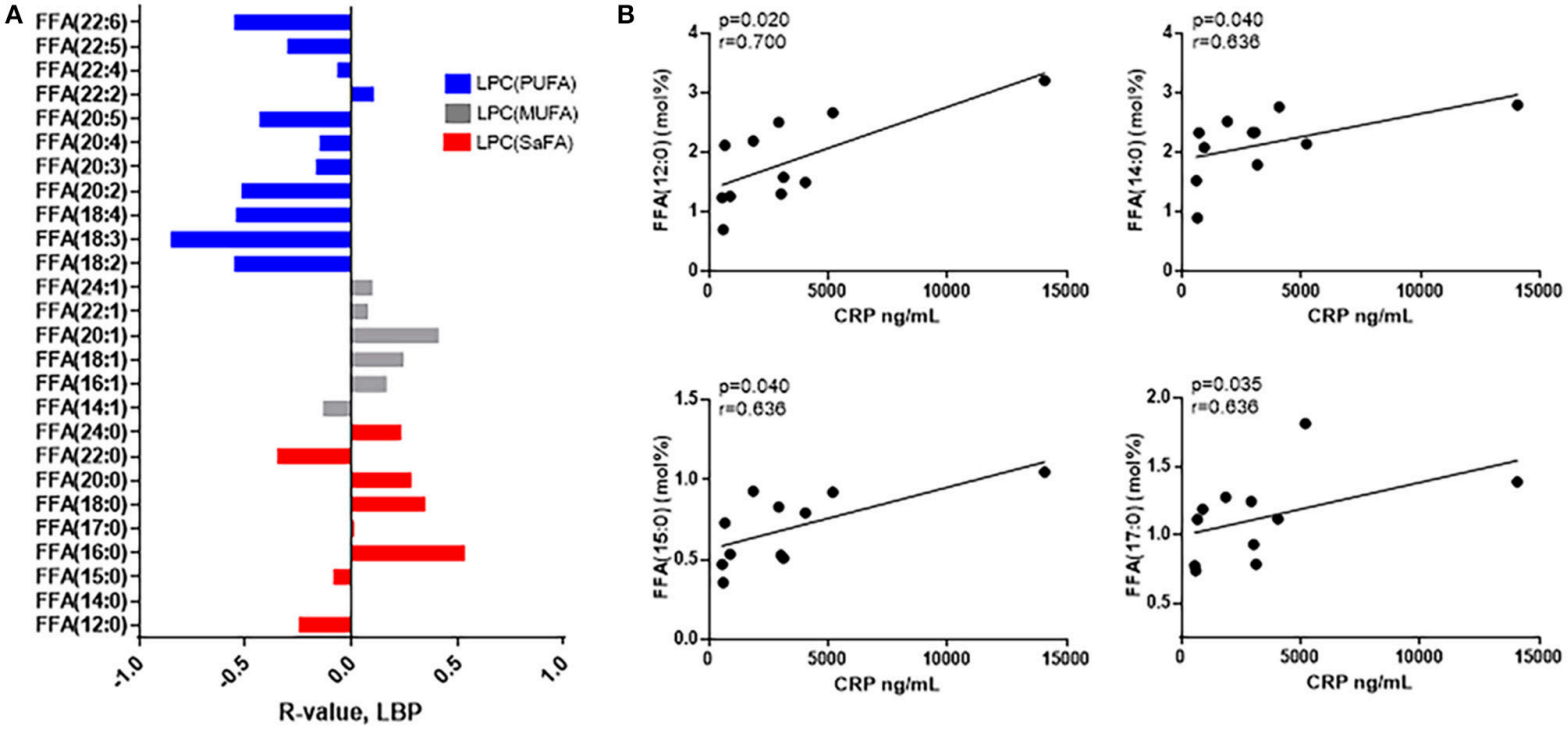
The composition of free fatty acid species is associated with markers of immune activation in HIV+ participants. **(A)** SaFAs (red) tend to be directly related, and PUFAs (blue) are inversely related to LBP in HIV+ participants. **(B)** CRP is directly associated with several free SaFA species in HIV+ study participants. Spearman correlation values are reported for these associations.

Immune activation persists in HIV+ individuals, even following virologic suppression with ART (18) (Supplemental Figures 2, 3). The composition of free fatty acids among HIV+ participants was related to serum levels of CRP and LBP, markers that are predictive of morbidity and mortality in HIV+ populations (23, 24). Overall, proportions of free/non-esterified SaFAs were directly associated with immune activation, and proportions of PUFAs were inversely associated with immune activation. HIV infection is associated with microbial translocation (25), and representative data for relationships among free fatty acids and LPS binding protein (LBP), a marker for microbial translocation, are shown in Figure 2A. While not all associations were statistically significant, by reporting each free fatty acid measured, the overall trends among the SaFAs and UFAs can be appreciated. Furthermore, the proportional amounts of the individual free SaFAs lauric acid (12:0), myristic acid (14:0), pentadecylic acid (15:0), and margaric acid (17:0) were positively associated with serum levels of CRP (*p* < 0.05 for all) (Figure 2B).

**Figure 3:**
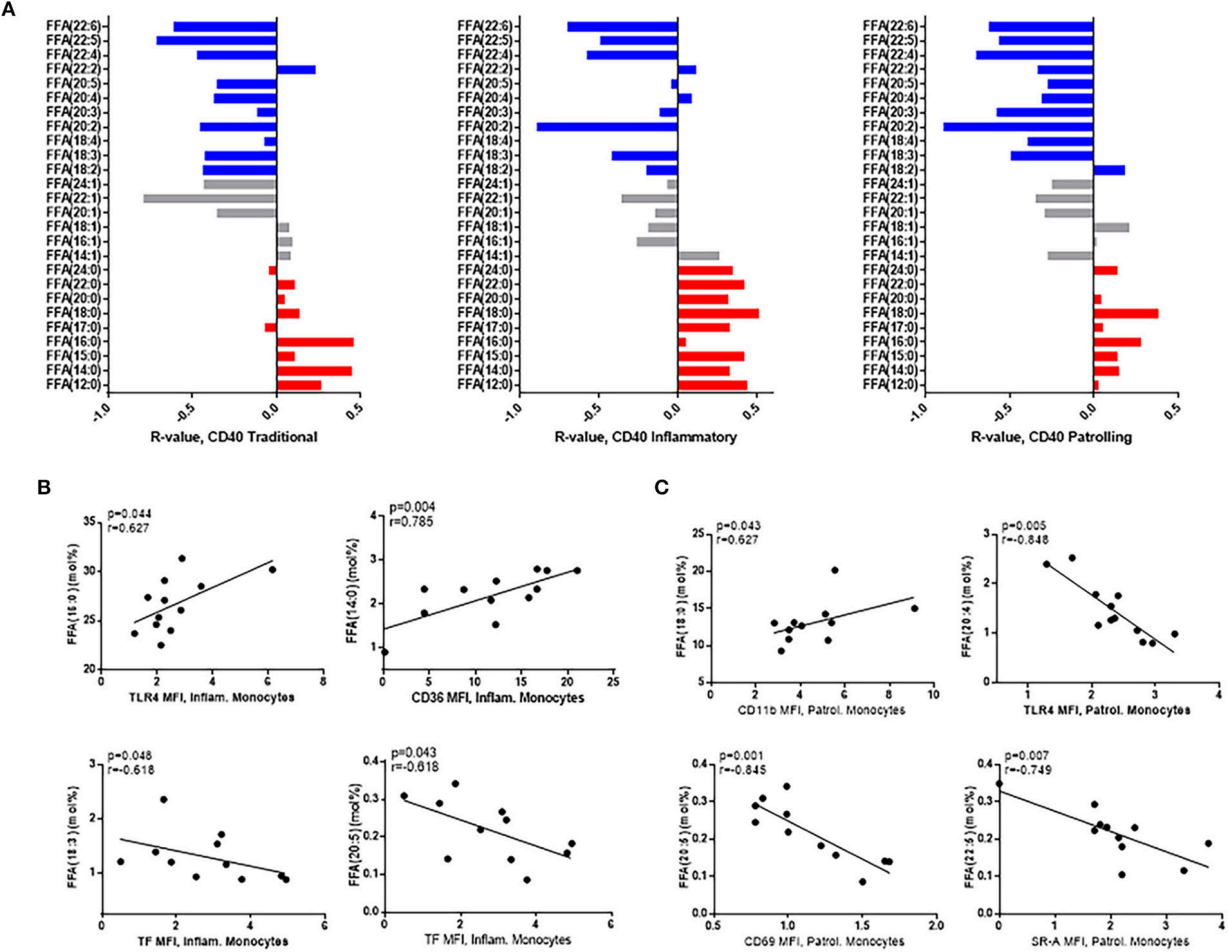
Free fatty acid composition is associated with monocyte activation in HIV+ participants. **(A)** SaFAs (red) are directly related, and PUFAs (blue) are negatively related to CD40 expression on monocyte subsets from HIV+ individuals. **(B)** Spearman correlations are reported for relationships among free fatty acid species and markers of inflammatory **(B)** and patrolling **(C)** monocyte activation in HIV+ individuals. Surface marker expression was measured by flow cytometry on monocyte subsets (identified by size, density, and expression levels of CD14 and CD16).

The composition of free fatty acids was also associated with monocyte activation markers, and several studies, including our own, have demonstrated an important role for monocytes in comorbid disease progression (26, 27). The surface expression of activation markers, CD40 and HLA-DR, and the adhesion molecule, CD11a, is increased on monocytes in HIV infection, and in general, the proportions of SaFAs were directly associated with expression of CD40 (Figure 3A) HLA-DR, and CD11a (Supplemental Figures 4A,B) on each monocyte subset, whereas the proportions of PUFAs were inversely associated with expression of these markers. Palmitic acid (16:0) levels were positively associated with inflammatory monocyte (CD14^+^CD16^+^) surface expression of the innate immune receptor, TLR4, and myristic acid (14:0) was positively associated with levels of CD36, a scavenger receptor involved in immune signaling and lipid uptake. Tissue factor (TF) is a pro-coagulant molecule previously shown to be related to immune activation in HIV infection (28), and TF surface expression on inflammatory monocytes was inversely related to the proportions of PUFAs α-linolenic acid (18:3) and EPA (20:5) (Figure 3B). Additionally, stearic acid (18:0) was directly associated with expression of the adhesion molecule CD11b on patrolling (CD14^dim^CD16^+^) monocytes. Also on patrolling monocytes, arachidonic acid (20:4) was inversely related to TLR4 expression, and EPA (20:5) was inversely related to CD69 expression (Figure 3C). Scavenger receptor-A (SR-A) is an important scavenger receptor for lipid uptake and innate immune signaling, and we report here, for the First time, that expression of SR-A is increased on monocyte subsets from HIV+ individuals compared to the monocyte expression in the HIV– study population (Supplemental Figure 3). SR-A levels were inversely related to levels of the PUFA, docosapentaenoic acid (DPA) (22:5) (Figure 3C). Additionally, among HIV+ participants, proportions of the PUFA α-linolenic acid (18:3) were positively associated with HDL (*r* = 0.53, *p* = 0.03) and CD4^+^ T-cell counts (*r* = 0.62, *p* = 0.004), and inversely related to TC (*r* = −0.48, *p* = 0.05) and TG levels (*r* = −0.56, *p* = 0.02) (Supplemental Figure 5A). Total levels of saturated FFAs were inversely associated with HDL levels and were directly associated with increased Framingham risk scores in the HIV+ study population (Supplemental Figure 5B).

### The Concentration and Fatty Acid Composition of LPC Is Altered in HIV Infection

The concentration of LPCs containing the PUFAs linoleic acid (18:2) and α-linolenic acid (18:3) was significantly decreased in the HIV+ group (*p* < 0.05). The composition of LPCs also differed between HIV+ and HIV– participants, with an overall enrichment of total SaFA-containing LPCs (64.3 vs. 60.7%, *p* = 0.1) and a reduction in the proportion of PUFA-containing LPCs (22.3 vs. 26.3%, *p* = 0.04) in the HIV+ participants (Figure 1B). There was a significant enrichment of LPC(17:0) and LPC(20:0), and LPC(18:0) tended to be increased in HIV+ individuals. We also measured significantly reduced proportional amounts of LPC (18:2) in HIV+ participants (Table 3). The serum concentration of total lysophosphatidylcholine (LPC) was not significantly different between HIV+ participants (300.5 μM) and HIV– participants (338 μM).

**Table 3:** Composition and concentration of LPC molecules measured in HIV– and HIV+ individuals.

	**Composition (mol %)**			**Concentration (μm)**		
**Lipid species**	**HIV–**	**HIV+**	***p*-value**	**HIV–**	**HIV+**	***p*-value**
LPC(14:0)	0.17	0.16	0.68	0.55	0.47	0.23
LPC(15:0)	0.31	0.32	0.77	1.03	0.95	0.45
LPC(16:0)	44.27	46.16	0.23	147.72	138.25	0.33
LPC(17:0)	**0.72**	**0.86**	**0.03**	2.40	2.52	0.51
LPC(18:0)	15.19	16.75	0.09	50.11	49.74	0.91
LPC(20:0)	**0.04**	**0.05**	**0.03**	0.13	0.14	0.59
LPC(16:1)	1.09	1.18	0.40	3.69	3.54	0.70
LPC(18:1)	11.86	12.16	0.61	40.51	37.02	0.37
LPC(20:1)	0.07	0.07	0.20	0.22	0.22	0.71
LPC(18:2)	**20.70**	**16.45**	**0.02**	**72.83**	**49.99**	**0.02**
LPC(18:3)	0.25	0.21	0.11	**0.85**	**0.62**	**0.04**
LPC(20:2)	0.17	0.17	0.78	0.57	0.51	0.35
LPC(20:3)	1.33	1.50	0.06	4.57	4.57	0.99
LPC(20:4)	3.40	3.51	0.68	11.83	10.58	0.37
LPC(20:5)	0.14	0.13	0.65	0.47	0.37	0.23
LPC(22:4)	0.04	0.04	0.27	0.14	0.13	0.78
LPC(22:5)	0.11	0.12	0.86	0.40	0.35	0.34
LPC(22:6)	0.16	0.18	0.41	0.55	0.51	0.62

*The bold values indicate p-values < 0.05*.

### LPC Fatty Acid Composition Correlates With Markers of Inflammation and Monocyte Activation

**Figure 4:**
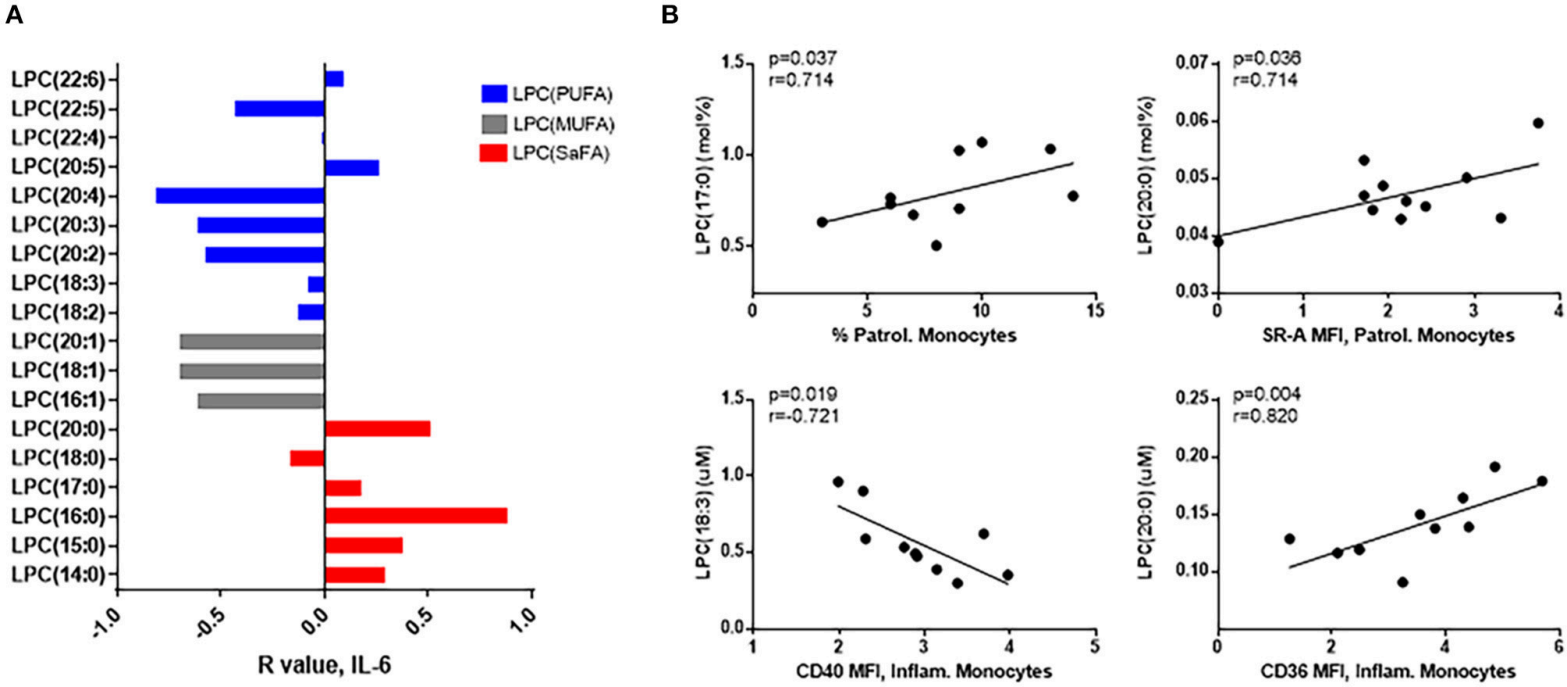
LPC molecules are associated with markers of monocyte and immune activation in HIV+ participants. **(A)** LPC species containing SaFAs (red) were positively associated, and LPC species containing MUFAs (gray) and PUFAs (blue) were negatively associated with serum levels of IL-6 in HIV+ individuals. **(B)** Spearman correlations are reported for relationships among LPC molecules and markers of monocyte activation in HIV+ individuals. Surface marker expression was measured by flow cytometry on monocyte subsets (identified by size, density, and expression levels of CD14 and CD16).

The composition of LPC species in HIV+ participants was related to immune activation. Overall, LPC species containing SaFAs were associated directly with serum levels of IL-6, and LPCs composed of monounsaturated fatty acids (MUFAs) and PUFAs were associated inversely with IL-6 (Figure 4A). LPCs enriched for the SaFA margaric acid (17:0) were positively associated with the proportion of patrolling monocytes, and LPC(20:0) was positively associated with surface expression of SR-A on patrolling monocytes and CD36 on inflammatory monocytes. LPC (18:3) was inversely associated with CD40 expression on inflammatory monocytes (Figure 4B).

LPC composition was also associated with traditional lipid measurements in HIV+ participants. Proportional amounts of LPC(15:0) were positively associated with both TC (*r* = 0.64, *p* = 0.004) and LDL levels (*r* = 0.52, *p* = 0.03). The proportion of PUFA-enriched LPC (22:6) was positively associated with HDL (*r* = 0.53, *p* = 0.02) and inversely associated with TG levels (*r* = −0.45, *p* = 0.05). Overall, LPC species enriched for MUFAs and PUFAs were positively related to CD4^+^ T-cell numbers, whereas SaFA-containing LPCs tended to be inversely related (Supplemental Figures 6A,B).

### Composition of Saturated and Unsaturated Free and LPC Fatty Acids Correlate With Markers of Immune Activation in HIV Uninfected Individuals

**Figure 5:**
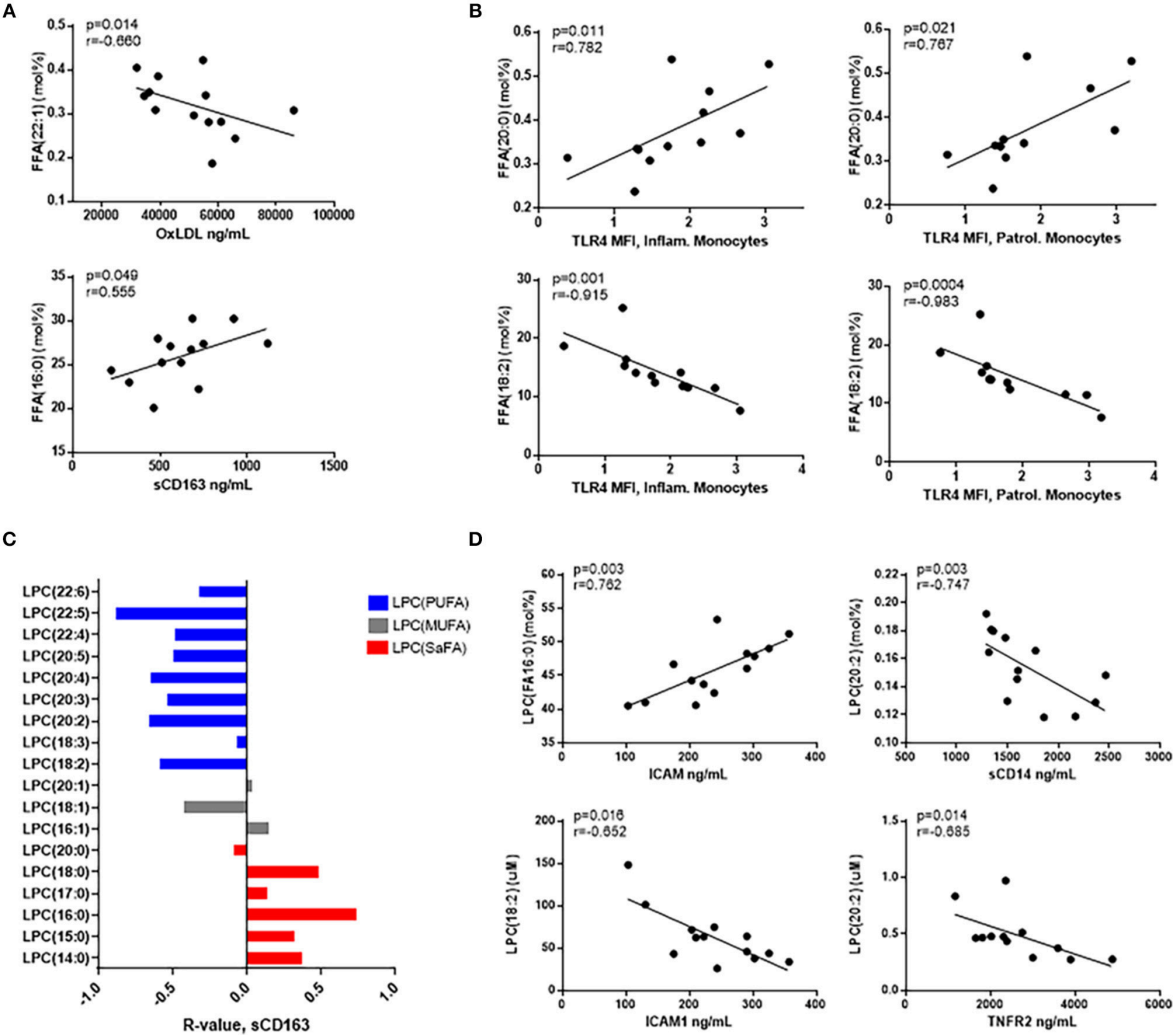
Free fatty acids and LPC molecules are also associated with markers of monocyte and immune activation in HIV- participants. **(A)** Spearman correlations are reported for relationships among free fatty acid species and serum immune activation markers and **(B)** monocyte activation marker, TLR4. **(C)** LPC species containing SaFAs (red) are directly related, and LPC species containing PUFAs (blue) are negatively related to serum levels of sCD163 in HIV– individuals. **(D)** Serum biomarkers of immune activation were inversely associated with PUFA-containing LPC species, and SaFA-containing LPC(16:0) was positively associated with ICAM1, a marker of endothelial cell activation.

Previously, lipidomic profiling has linked specific lipid species to CVD risk in HIV– individuals (29–31). Therefore, we investigated whether the proportional representation of fatty acids among free and LPC molecules were associated with markers of inflammation in our HIV– participants. Free palmitic acid (16:0) was positively associated with serum levels of sCD163, and free erucic acid (22:1) was inversely related to serum levels of oxidized LDL (Figure 5A). Free arachidic acid (20:0) was significantly positively associated with surface expression of TLR4 on inflammatory and patrolling monocytes, whereas free linoleic acid (18:2) was inversely associated with TLR4 expression on these cells (Figure 5B).

Proportional amounts of LPC(16:0) were positively associated with serum levels of ICAM1, and LPC(18:2) was negatively associated with ICAM1. We also measured inverse relationships between LPC(20:2) and levels of sCD14 and TNFR2 in HIV– participant serum (Figure 5D). Additionally, SaFA-enriched LPC was overall directly related to sCD163 measurements, and PUFA-enriched LPC was inversely related to sCD163 (Figure 5C). These data suggest, while greater perturbations in the lipidome in HIV+ individuals are associated with increased levels of immune activation; associations among SaFAs, PUFAs, and markers associated with inflammation and CVD risk are also present in HIV-uninfected individuals.

### Saturated Free Fatty Acid Exposure Alters Monocyte Subset Proportions and Activation *in vitro*

**Figure 6:**
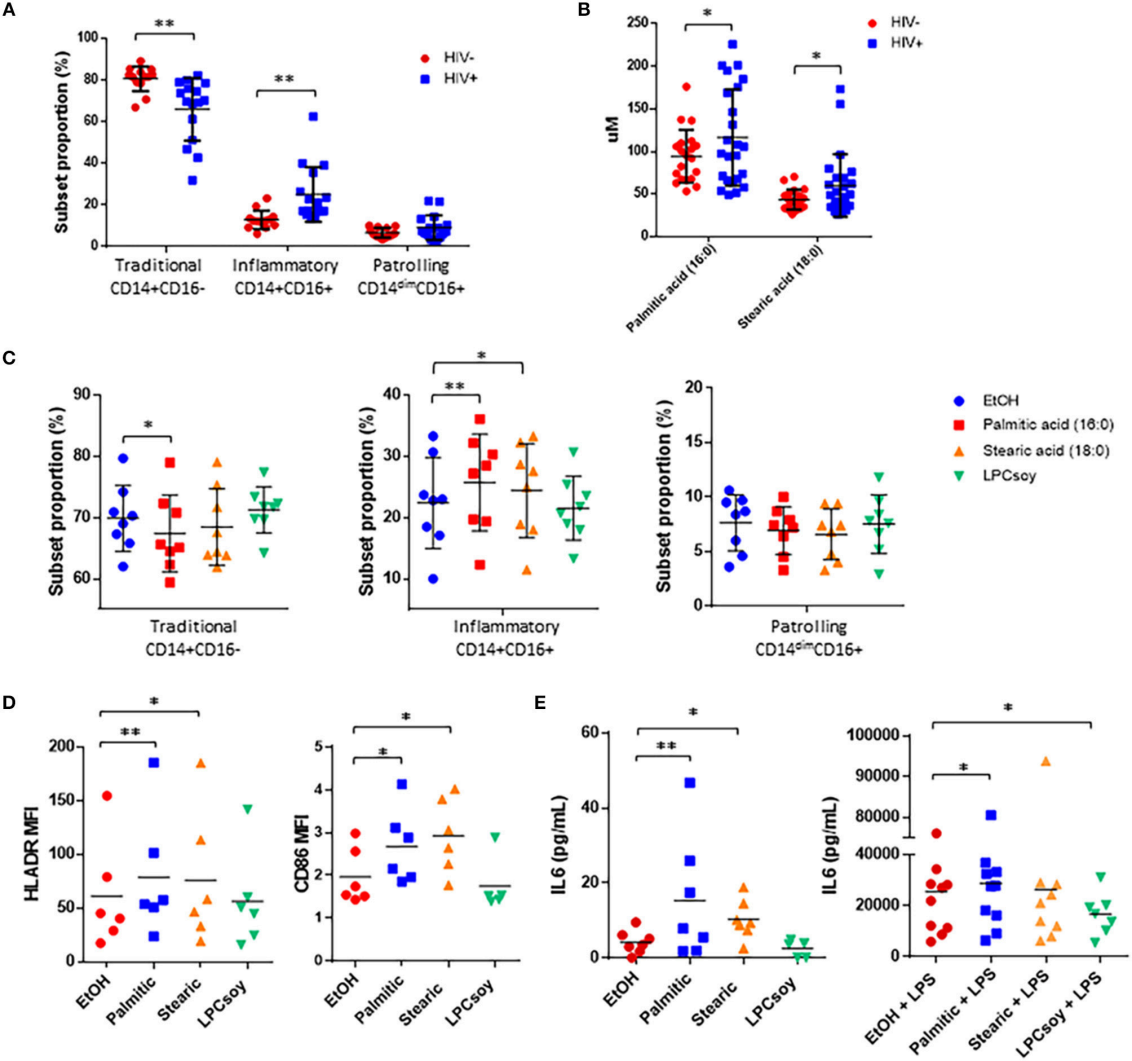
Free fatty acid exposure alters monocyte subset proportion and activation, and inflammatory cytokine production *in vitro***. (A)** Summary data of monocyte subset proportion measured in HIV– and HIV+ participants. **(B)** Serum concentration of palmitic acid (16:0) and stearic acid (18:0) measured in HIV– and HIV+ participants. **(C)** Whole blood obtained from HIV– donors was exposed for 3 h to ethanol (EtOH) vehicle control, palmitic acid (16:0), stearic acid (18:0), or LPC enriched for PUFAs (LPCsoy) (10 uM for all). Cells were analyzed by flow cytometry and monocyte subset proportions were determined based on surface expression of CD14 and CD16. PBMCs were exposed to the indicated fatty acids for 24 h, and monocyte activation markers were measured by **(D)** flow cytometry and supernatant expression of IL-6 was measured by **(E)** ELISA. ^*^p < 0.05, ^**^p < 0.01.

Alterations in the proportional representation of monocytes in HIV infection has been reported previously (32) and here, we also measured a significant increase in the proportion of inflammatory (CD14^+^CD16^+^) monocytes, and patrolling (CD14^dim^CD16^+^) monocytes in HIV+ participants. There was a concomitant decrease in the proportion of traditional (CD14^+^CD16^−^) monocytes in HIV+ participants compared to HIV– participants (Figure 6A, Supplemental Figure 1).

The concentrations of the free SaFAs palmitic acid (16:0) and stearic acid (18:0) were increased significantly in the HIV+ group (Figure 6B) and proportions of inflammatory monocytes were positively associated with levels of both palmitic acid (16:0) (*r* = 0.72, *p* = 0.02) and stearic acid (18:0) (*r* = 0.64, *p* = 0.05), whereas proportions of traditional monocytes were negatively associated with stearic acid (18:0) (*r* = −0.86, *p* = 0.002) (data not shown).

To determine whether palmitic acid (16:0) or stearic acid (18:0) exposure affects monocyte subset proportions *in vitro*, we exposed whole blood from HIV– donors to these free fatty acids for 3 h. We measured a significant increase in the proportion of inflammatory monocytes and a reduction in the proportion of traditional monocytes stimulated with palmitic acid (16:0), and significantly increased inflammatory monocytes in blood stimulated with stearic acid (18:0). Exposure of whole blood to PUFA-enriched LPC (isolated from soybeans, LPCsoy) did not alter monocyte subset phenotype compared to ethanol treated controls (Figure 6C).

We next measured monocyte activation marker expression following overnight exposure of HIV– donor PBMCs to palmitic acid (16:0) and stearic acid (18:0), and observed significantly increased expression of HLA-DR and CD86 on monocytes stimulated with free SaFAs (Figure 6D). LPCsoy exposure did not increase expression of monocyte activation markers. PBMCs also produced more IL-6 following exposure to palmitic acid (16:0) and stearic acid (18:0). Lastly, palmitic cid (16:0) enhanced, while LPCsoy significantly inhibited, LPS-induced production of IL-6 from PBMCs (Figure 6E).

## Discussion

Both HIV infection and its treatment can alter lipid profiles, decreasing levels of HDL, and increasing levels of LDL and TC (1, 2). Traditional lipid panels, however, may not adequately evaluate CVD risk in HIV infection (4). Previous work, including our own, has implicated free fatty acids and LPCs as important for the pathogenesis of CVD (12, 33–35). Here, we compared the concentration and composition of free and LPC fatty acid species measured in HIV+ individuals taking suppressive ART and HIV– individuals, and explored relationships among these lipids and monocyte and immune activation markers.

We demonstrated several differences in the lipidomes of our participants, including significantly elevated total free fatty acid levels in HIV+ participant serum, despite similarities in traditional lipid panels (i.e., LDL, HDL, TC) between the groups. Lipidomic profiling has outperformed conventional lipid panels to predict future CVD in an HIV– population, and may improve cardiovascular risk screening of HIV+ individuals as well (4, 29, 36). Increased levels of circulating free fatty acids have been linked with obesity (33), insulin resistance (37), and cardiovascular disease risk (33, 34). We also observed an enrichment of free SaFAs and SaFA-containing LPC species from HIV+ participants; SaFAs promote inflammation (14), and may contribute to chronic immune activation and the development of comorbidities in HIV infection. Total free SaFA levels directly correlated with Framingham 10-year CVD risk calculations in this HIV+ study population and though lipid levels contribute to this traditional risk score, as noted above, more detailed characterization of lipid species may offer better prediction of risk than levels of HDL that contribute to Framingham risk Here, we report several direct relationships among SaFAs and markers of inflammation and immune activation, including relationships among SaFAs and increased levels of CRP.

In addition, we measured decreased proportional amounts of free PUFAs and PUFA-containing LPC species in our HIV+ population. Unsaturated fatty acids may inhibit inflammation (12, 15–17); accordingly, we identified several inverse relationships between PUFAs, and monocyte activation and inflammatory markers in HIV+ participants. PUFA levels may also protect against the development and progression of diabetes, obesity, NAFLD, and NASH (8–11). Depletion of PUFAs has been associated with hepatic triglyceride accumulation, endoplasmic reticulum stress, and unfolded protein responses (38, 39). By interacting with PPAR transcription factors, PUFAs modulate fatty acid oxidation pathways and inflammatory mediators, NF-kB, AP-1, NFAT, and STATs, in macrophages and lymphocytes (40). The PUFAs, EPA (20:5) and DHA (22:6), can inhibit TLR signaling and inflammasome activation (15), whereas SaFAs, including stearic acid (18:0), activate the inflammasome and trigger IL-1β release in myeloid cells (14). In a randomized placebo-controlled trial, oral supplementation of EPA (20:5) and DHA (22:6) reduced inflammation and soluble TNFR1 levels in HIV+ individuals (41). Dyslipidemia and imbalanced proportions of SaFAs and PUFAs may contribute to chronic inflammation and directly alter progression of diseases, including CVD and HIV infection.

The intestinal microbiome plays important roles in regulating metabolism of dietary lipids (42). Microbial dysbiosis and increased plasma levels of microbial products have been observed in HIV+ individuals, and these indices relate to immune activation (25). In mice, increased PUFA levels alter microbiome composition resulting in decreased LPS producing bacteria and improved gut barrier function (43). In our HIV+ population, serum levels of free PUFAs were inversely related to LPS-binding protein (LBP), whereas free SaFAs were directly related, suggesting a potential link between lipids and microbial translocation in HIV infection. Additionally, *in vitro* exposure of PBMCs to palmitic acid (16:0) enhanced, whereas PUFA-enriched LPC treatment significantly inhibited LPS-induced IL-6 production, these findings suggest HIV-associated altered lipid profiles may exacerbate the inflammatory consequences of microbial translocation.

Increased levels of total LPC concentrations have been observed in CVD (12) and we recently reported elevated LPCs in ART-naïve HIV+ individuals at baseline and following 48 weeks of treatment with a Raltegravir-based regimen, compared to levels in an HIV– population (35). In this current study, we did not detect a significant difference in total LPC concentrations between our HIV- and ART-treated HIV+ populations. We did, however, measure increased LPC species enriched for SaFAs, including LPC(17:0) and LPC(20:0), whereas the proportional amount of LPC(18:2) was significantly decreased. The fatty acid composition of LPC molecules is a major functional determinant; SaFA-containing LPCs tend to have pro-atherogenic properties, and conversely, PUFA-containing LPCs tend to be anti-atherogenic (12). In HIV+ participants, we report that SaFA-containing LPC species were directly associated, while PUFA-containing LPC species were inversely associated, with levels of IL-6. Levels of PUFA containing LPCs were also directly related to CD4 counts. Interleukin-6 and CD4 T cell counts have been linked to morbidity in HIV infection (44, 45). Notably, none of the current HIV+ study participants was taking Raltegravir, and the overall duration of ART usage was longer than in our previous study, potentially allowing for further changes in certain lipid levels. Moreover, 60% of this population were statin users, compared to only 9% statin usage in the HIV+ population from our previous study, which may also account for some variation in total lipid concentrations between the two studies. We did not observe significant associations among FFA levels and statin use, however levels of LPC(14:0) were significantly reduced in statin users (0.39 μM) compared to non-users (0.56 μM) (*p* = 0.04).

We also observed several associations among free fatty acids and LPC species and markers of monocyte activation in HIV– participants. Activated monocytes and macrophages have been implicated in progression of atherosclerosis in HIV– and HIV+ populations. Overall, free SaFA species tended to correlate directly with monocyte surface expression of CD40, HLA-DR, and CD11a. Previously, we have reported increased levels of the adhesion molecule CD11a on monocyte subsets in HIV infection, potentially enhancing the vascular homing capabilities of these cells (46). Increased linoleic acid (18:2) concentration has been previously linked to reduced CVD risk (47), and in our HIV– population this free fatty acid was inversely related to TLR4 expression on both inflammatory (CD14^+^CD16^+^) and patrolling (CD14^dim^CD16^+^) monocytes. Studies have implicated an important role for TLR4 in metabolic inflammation; long chain SaFAs can activate TLR4 signal transduction and promote inflammatory cytokine expression and insulin resistance (48, 49). Additionally, serum levels of soluble CD163, a marker of monocyte activation previously linked to CVD risk and arterial inflammation (50, 51), were directly related to SaFA-containing LPC species, and inversely related to PUFA-containing LPC species. The composition of free PUFAs, including levels of α-linolenic acid (18:3) and EPA (20:5), were inversely associated with tissue factor (TF) expression on inflammatory monocytes. Monocytes from HIV+ individuals often express high levels of TF (28), and TF can initiate the extrinsic clotting pathway (52), contributing to a potentially pro-thrombotic state.

The associations among fatty acid levels, inflammation and monocyte activation may be mechanistically important, as we also report that *in vitro* exposure of PBMCs from uninfected donors to the SaFAs palmitic acid (16:0) and stearic acid (18:0) resulted in increased monocyte activation marker expression and IL-6 production. Further, SaFA exposure increased the proportional representation of inflammatory (CD14^+^CD16^+^) monocytes. Inflammatory monocytes produce pro-inflammatory cytokines, reactive oxygen species (ROS), and express vascular homing molecules, including CX3CR1 and CD11a, indices that have been associated with the development of atherosclerosis (53). Increased proportions of inflammatory monocytes have been observed in HIV infection (32), sepsis (54), and coronary artery disease (27). Since circulating monocytes can migrate into the subendothelial space and differentiate into macrophages that contribute to vascular inflammation and atherosclerotic plaque formation (55), altered circulating monocyte phenotype may have important implications for the migration of monocytes to the vascular endothelium and subsequent differentiation and polarization of macrophages. Further examination of the relationship between altered lipid profiles in HIV infection and macrophage phenotype and function is warranted.

Our cross sectional study has limitations. Our HIV– and HIV+ groups were not well matched for age due to recruitment limitations. Participant age, however, did not statistically correlate with levels of any of the free fatty acid species described in this study. We were unable to adjust for dietary intake in our study participants, and diet likely has important effects on the overall lipidome. In HIV+ participants, we did not observe any significant associations between BMI and the concentration or composition of LPC molecules, however, BMI was inversely associated with the concentration of free PUFAs, α-linolenic acid (18:3) (*r* = −0.542, *p* = 0.025) and DHA (22:6) (*r* = −0.539, *p* = 0.026). Dietary assessments and information on body composition and metabolic hormone status should be included in future studies. The HIV– and HIV+ study groups had similar levels of TC, HDL, LDL, and TG, and yet, were distinguishable by examining specific lipid moieties. Here we identified broad differences in the detailed lipidome, highlighting the limitations of traditional lipid measurements for risk assessment. Another limitation to our study is that we did not control for specific ART regimens. HIV-associated dyslipidemia has been observed prior to the advent of ART (1), however, ART can also alter lipid metabolism, possibly contributing to increased risk of atherosclerosis and CVD (56). ART-associated lipid abnormalities are most evident with protease inhibitor use, particularly when boosted by inhibitors of cytochrome p450 3A4, although other drug classes, including non-nucleoside reverse transcriptase inhibitors (NNRTIs), also have relevant effects on lipid levels (56–58). The ALTAIR trial, in which treatment-naïve HIV+ individuals were randomized to one of three initial ART regimens (efavirenz-, ritonavir-boosted atazanavir-, or zidovudine/abacavir-based regimens), demonstrated differential effects of ART regimens on the lipidome (59).

Despite its limitations, our study may be an important resource in mapping out strategies to prevent the development of comorbidities in HIV+ and HIV– populations. We describe significant associations among lipids and markers of monocyte and immune activation within HIV– and HIV+ populations, and report altered proportions of SaFAs and PUFAs in HIV+ individuals that may exacerbate inflammation and disease progression. Dyslipidemia may be both a consequence and a driver of immune activation in HIV infection. Analyses of the altered lipidome will likely improve our ability to predict CVD risk in HIV+ individuals, and identify novel lipid biomarkers with greater prognostic value than conventional lipid measurements. Further work is needed to investigate the complex and in-depth interactions among the lipidome, immune activation, specific ART regimens, and morbidity and mortality in ART-treated HIV infection.

## Ethics Statement

All experiments were approved by the Institutional Review Board at Ohio State Wexner Medical Center. Participants provided samples following informed consent.

## Author Contributions

All authors contributed to experimental design, data analysis, and writing of the manuscript. EB, MK, JG, KR, and MJC: performed experiments. BR, CC, and MC: provided analytical expertise.

### Conflict of Interest Statement

NF has served as a paid consultant for Gilead Science Inc. ML has received grant support from Gilead Sciences; JL has served as a paid consultant to Gilead and Merck, and has received grant support from Gilead Sciences. The remaining authors declare that the research was conducted in the absence of any commercial or financial relationships that could be construed as a potential conflict of interest.

## Supplementary Material

Supplemental Figure 1 Monocyte subsets were identified by **(A)** size and granularity, and by **(B)** surface expression of CD14 and CD16. Representative dot plots indicate the gating strategy used to identify monocyte subsets, and demonstrate differences in monocyte subset proportions from **(C)** 1 HIV– and **(D)** 1 HIV+ study participant.

Supplemental Figure 2 Serum biomarkers of inflammation (TNFR-2, IL-6, CRP), monocyte (sCD14, sCD163) and endothelial cell (ICAM1, VCAM1) activation, and microbial translocation (LBP) are increased in ART-treated HIV-infection. Analytes were measured by ELISA. ^*^*p* < 0.05, ^**^*p* < 0.005, ^***^*p* < 0.001.

Supplemental Figure 3 **(A)** The scavenger receptor, SR-A, was measured by flow cytometry, and representative histograms for SR-A expression on monocyte subsets from HIV– and HIV+ study participants are shown. **(B)** Summary data of SR-A expression on monocyte subsets (% positive cells) from HIV– and HIV+ study participants. **(C)** Activation marker expression differs on monocyte subsets from HIV– and HIV+ individuals. Shown here are comparative summary data for mean fluorescence intensity (MFI) averages of monocyte subset surface markers (CD11a, CD11b, CD40, TLR4, HLA-DR, CD69, TF, CD36, SR-A). *P*-values were calculated using Mann-Whitney testing. ^*^*p* < 0.05.

Supplemental Figure 4 The composition of free fatty acids is associated with monocyte activation in HIV+ participants. **(A)** SaFAs (red) tend to be directly related, and PUFAs (blue) are inversely related to HLADR and **(B)** CD11a in HIV+ participants.

Supplemental Figure 5 The composition of free fatty acids is associated with traditional lipid profiles and clinical indices in HIV+ participants. Spearman correlations are reported for relationships among **(A)** proportions of the free PUFA species, α-linolenic acid (18:3), and basic clinical measurements, and **(B)** total saturated FFA concentration and Framingham risk scores and HDL levels (mg/dL).

Supplemental Figure 6 The composition of LPC molecules is associated with traditional lipid profiles and clinical indices in HIV+ participants. **(A)** SaFAs (red) tend to be inversely related, and PUFAs (blue) are directly related to CD4+ T-cell numbers. **(B)** Spearman correlations are reported for relationships among proportions of LPC species and traditional lipid measurements.
