# Supplementary figures and images for: Altered Lipidome Composition Is Related to Markers of Monocyte and Immune Activation in Antiretroviral Therapy Treated Human Immunodeficiency Virus (HIV) Infection and in Uninfected Persons
Source: Front Immunol. 2019 Apr 16;10:785. doi: 10.3389/fimmu.2019.00785 (PMC6477036; doi:10.3389/fimmu.2019.00785)

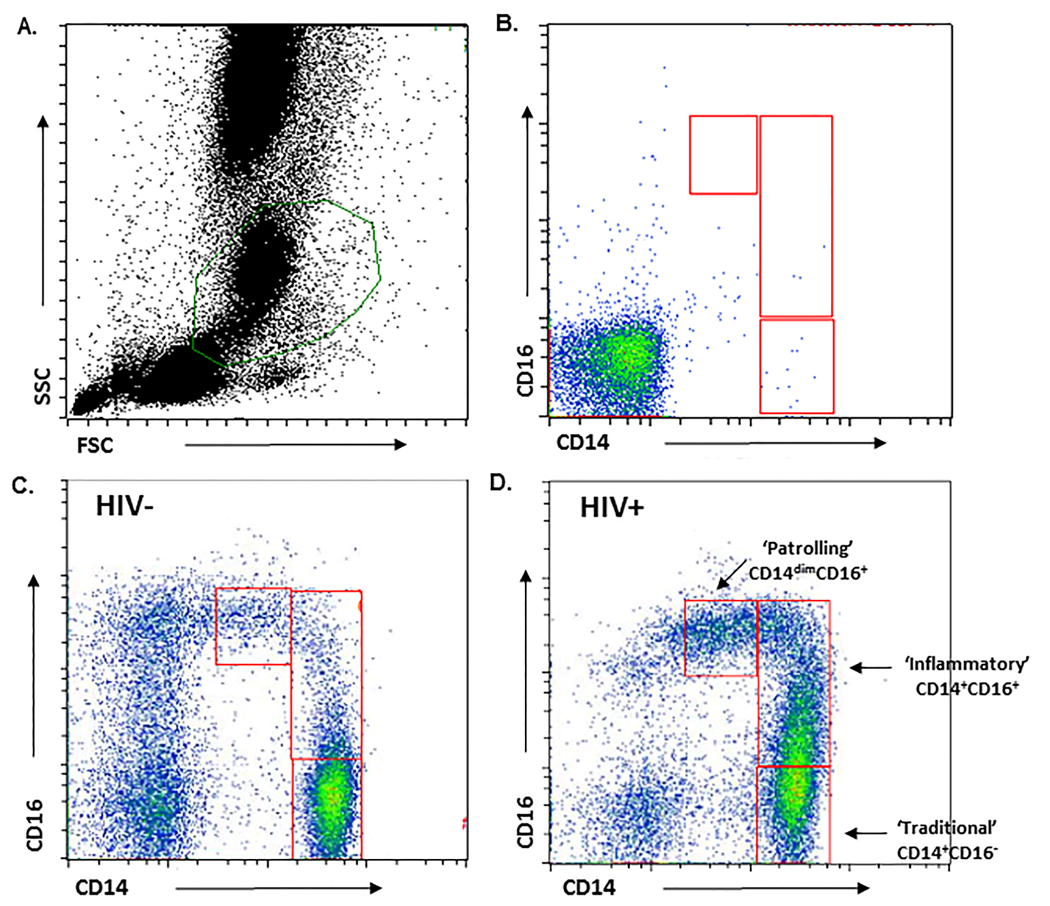

Supplement: Supplemental Figure 1 — Monocyte subsets were identified by (A) size and granularity, and by (B) surface expression of CD14 and CD16. Representative dot plots indicate the gating strategy used to identify monocyte subsets, and demonstrate differences in monocyte subset proportions from (C) 1 HIV– and (D) 1 HIV+ study participant. [file Image_1.TIF]

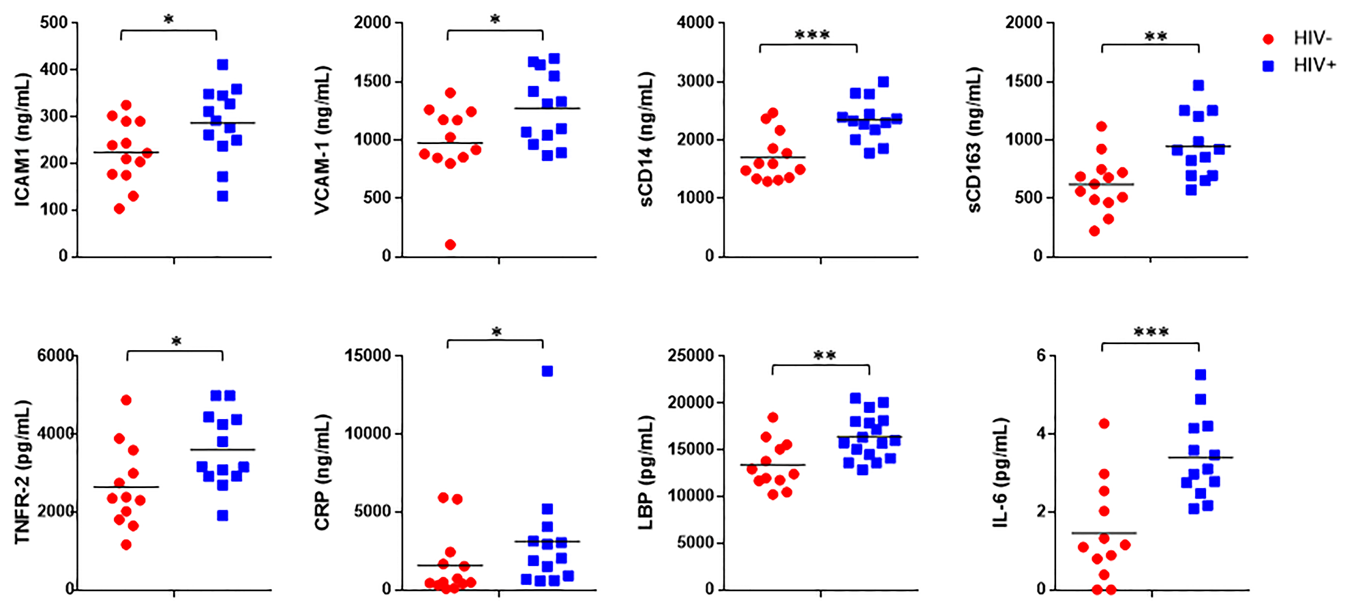

Supplement: Supplemental Figure 2 — Serum biomarkers of inflammation (TNFR-2, IL-6, CRP), monocyte (sCD14, sCD163) and endothelial cell (ICAM1, VCAM1) activation, and microbial translocation (LBP) are increased in ART-treated HIV-infection. Analytes were measured by ELISA. *p < 0.05, **p < 0.005, ***p < 0.001. [file Image_2.TIF]

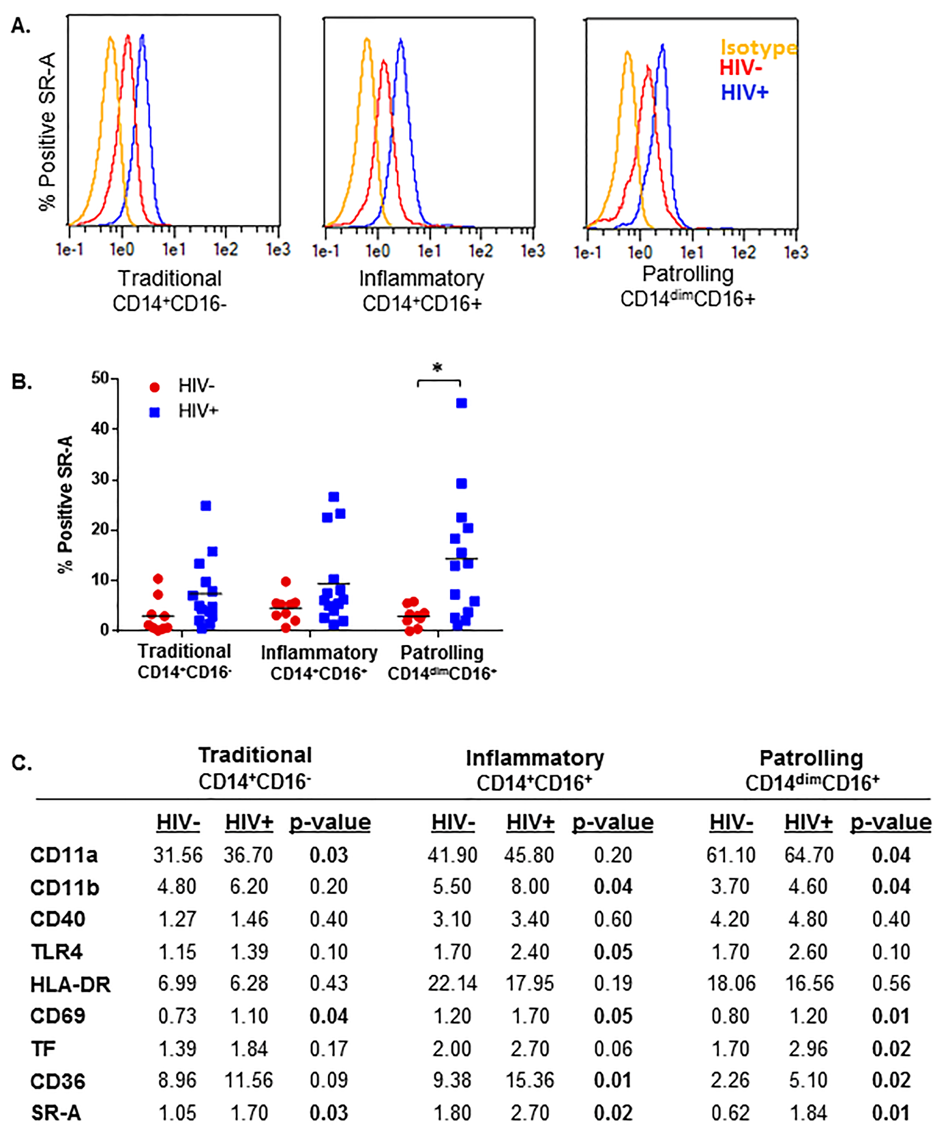

Supplement: Supplemental Figure 3 — (A) The scavenger receptor, SR-A, was measured by flow cytometry, and representative histograms for SR-A expression on monocyte subsets from HIV– and HIV+ study participants are shown. (B) Summary data of SR-A expression on monocyte subsets (% positive cells) from HIV– and HIV+ study participants. (C) Activation marker expression differs on monocyte subsets from HIV– and HIV+ individuals. Shown here are comparative summary data for mean fluorescence intensity (MFI) averages of monocyte subset surface markers (CD11a, CD11b, CD40, TLR4, HLA-DR, CD69, TF, CD36, SR-A). P-values were calculated using Mann-Whitney testing. *p < 0.05. [file Image_3.TIF]

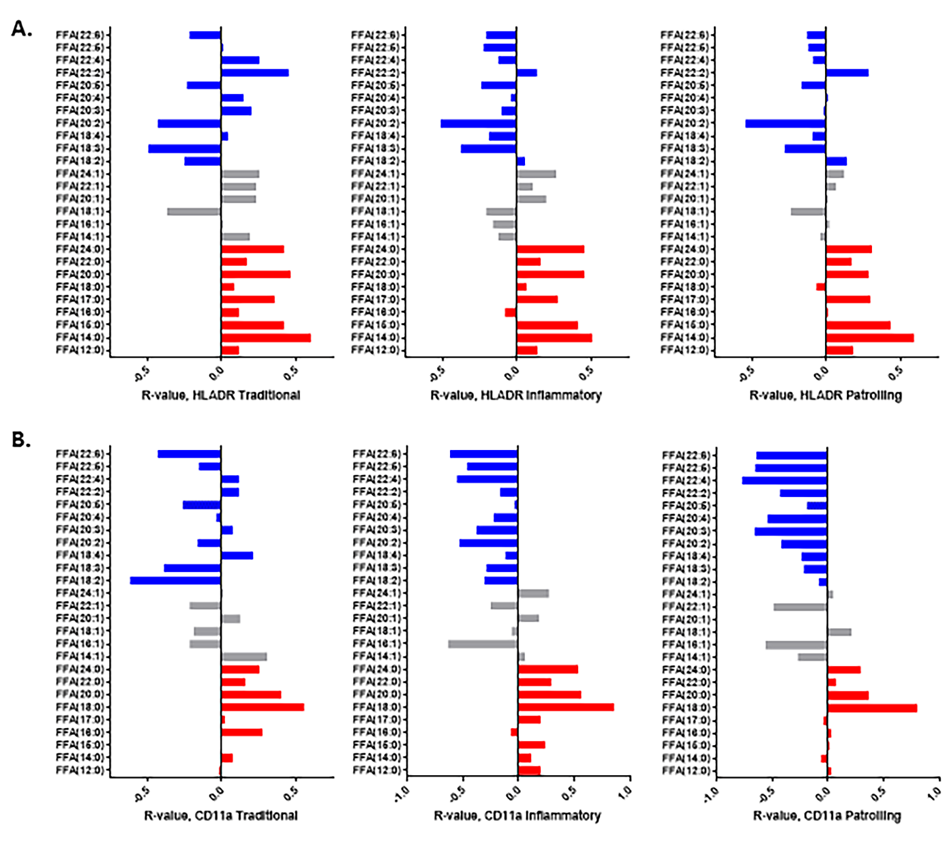

Supplement: Supplemental Figure 4 — The composition of free fatty acids is associated with monocyte activation in HIV+ participants. (A) SaFAs (red) tend to be directly related, and PUFAs (blue) are inversely related to HLADR and (B) CD11a in HIV+ participants. [file Image_4.TIF]

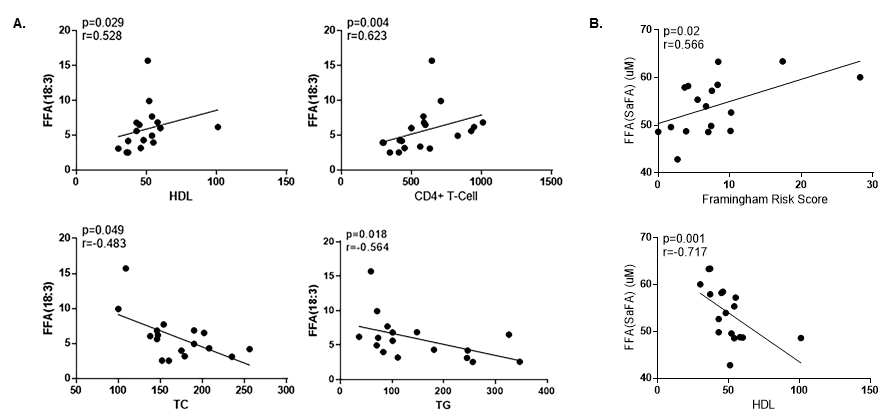

Supplement: Supplemental Figure 5 — The composition of free fatty acids is associated with traditional lipid profiles and clinical indices in HIV+ participants. Spearman correlations are reported for relationships among (A) proportions of the free PUFA species, α-linolenic acid (18:3), and basic clinical measurements, and (B) total saturated FFA concentration and Framingham risk scores and HDL levels (mg/dL). [file Image_5.TIF]

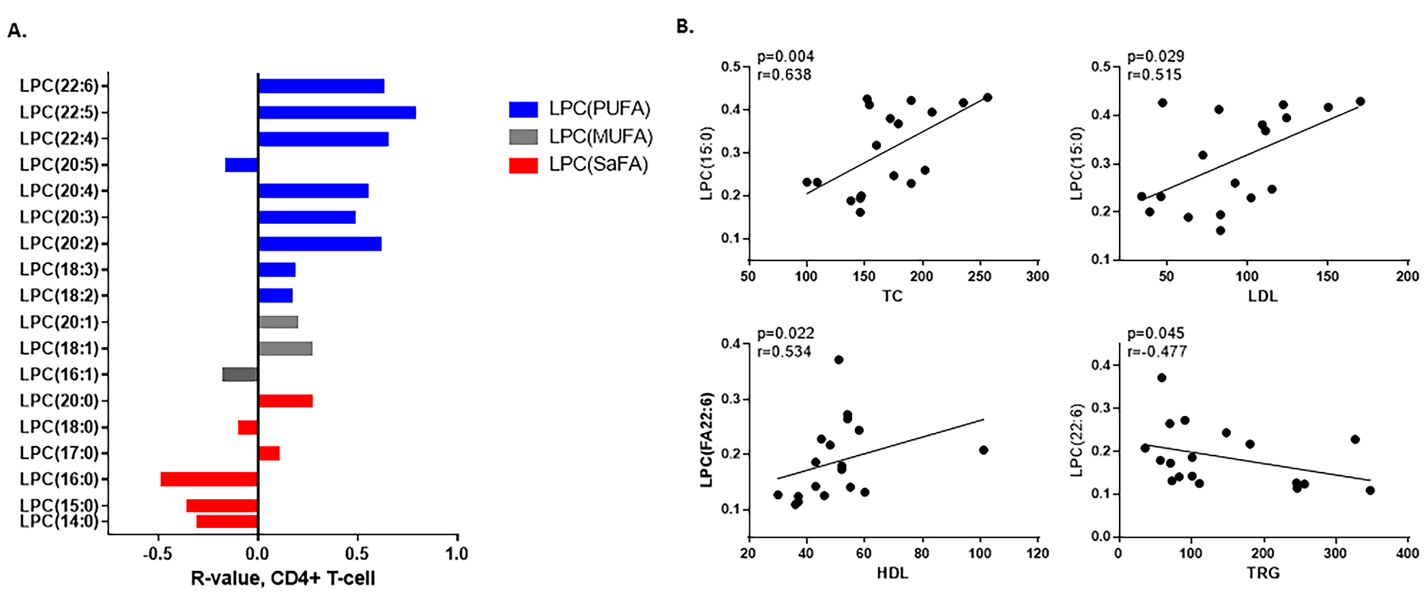

Supplement: Supplemental Figure 6 — The composition of LPC molecules is associated with traditional lipid profiles and clinical indices in HIV+ participants. (A) SaFAs (red) tend to be inversely related, and PUFAs (blue) are directly related to CD4+ T-cell numbers. (B) Spearman correlations are reported for relationships among proportions of LPC species and traditional lipid measurements. [file Image_6.TIF]
